# Ulcerative Colitis Presented as Fever and Bloody Diarrhea at Initiation of Dialysis in an Elderly Patient with End-Stage Kidney Disease

**DOI:** 10.1155/2015/725205

**Published:** 2015-11-04

**Authors:** Shunsuke Yamada, Yuka Kanazawa, Noriko Nakamura, Kosuke Masutani, Motohiro Esaki, Takanari Kitazono, Kazuhiko Tsuruya

**Affiliations:** ^1^Department of Medicine and Clinical Science, Graduate School of Medical Sciences, Kyushu University, Fukuoka 812-8582, Japan; ^2^Department of Integrated Therapy for Chronic Kidney Disease, Graduate School of Medical Sciences, Kyushu University, Fukuoka 812-8582, Japan

## Abstract

Ulcerative colitis (UC) is a chronic inflammatory bowel disorder that mainly affects the colon and rectum. Immunological derangements are associated with the pathogenesis of UC. Many patients with UC also have chronic kidney disease, associated with immunological disorders and/or pharmacotherapy for UC. Some patients with UC may develop end-stage renal disease (ESRD) and require renal replacement therapy. However, little is known clinically about ESRD patients who develop UC or about patients with UC who develop ESRD. This report describes an elderly patient with ESRD who presented with fever and bloody diarrhea and was finally diagnosed as UC (pancolitis type) at dialysis initiation. The patient was successfully treated with a series of immunosuppressive agents. This report highlights the importance of considering UC as a potential cause of bloody stool and fever in patients with ESRD.

## 1. Introduction

Ulcerative colitis (UC) is an inflammatory bowel disorder characterized by continuous colonic mucosal inflammation that extends proximally from the rectum [1]. The symptoms and signs of UC include abdominal pain, bloody diarrhea, urgency, and tenesmus, with clinical course depending on disease activity [2]. Patients may present with weight loss and other symptoms, such as low grade fever. Mounting evidence has revealed that the development of UC is mainly caused by bowel inflammation mediated by disordered humoral and cellular immunity. Accordingly, agents that modulate immunological derangements including sulfasalazine and steroids and biological agents are theoretically ideal and effective [3]. Clinical studies have also shown that environmental and genetic factors are also related to the development of UC [1, 2].

Patients with UC often develop nephritis, acute kidney injury, and chronic kidney disease (CKD) [4, 5]. Some patients with UC and CKD may develop end-stage renal disease (ESRD) and require dialysis therapy. Conversely, dialysis patients may develop UC. However, little is known about patients with both ESRD and UC or about patients who develop UC after the initiation of dialysis therapy. This report describes a patient with ESRD who presented with fever and bloody diarrhea at the initiation of dialysis. This patient was finally diagnosed with UC and was successfully treated with a combination of immunosuppressive agents.

## 2. Case Report

A 76-year-old woman with ESRD undergoing peritoneal dialysis (PD) treatment was admitted to our hospital because of persistent fever and mucous and bloody stool. She had been diagnosed with proteinuria at the age of 26 and developed pregnancy-induced hypertension at the age of 30. At the age of 62, she was diagnosed with CKD due to chronic glomerulonephritis and was periodically followed up for this condition. At the age of 76, she developed ESRD and was started on hemodialysis treatment and was subsequently transferred to PD treatment; at this time, she developed a fever (37-38°C). Physical examination and imaging, including a colonoscopy, could not reveal the origin of fever. She was suspected of having extrapulmonary tuberculosis, which is frequent in ESRD patients at the initiation of dialysis. Actually, tuberculin skin test and interferon-gamma releasing assay were positive, indicating that she was suffering from patent or active tuberculosis. Although we could not detect the site of active lesion related to tuberculosis, she was started on treatment with isoniazid, streptomycin, and rifampicin and was discharged from the hospital. However, while on isoniazid, her fever fluctuated. Two months after starting PD treatment, she developed sustained bloody diarrhea with progressive anemia. Because she was suspected of having active inflammatory bowel disease on colonoscopy, she was transferred to our hospital for further evaluation.

On admission, she was alert. Her blood pressure was 134/66 mmHg (supine position), her heart rate was 72 beats per minute, her respiration rate was 12/min, and her body temperature was 39.1°C. Physical examination showed that the palpebral conjunctiva was anemic but not icteric. Bowel sounds had increased. Tenderness was observed all over the abdomen. The exit of a peritoneal catheter was present in the left upper abdominal quadrant. Moderate pretibial pitting edema was observed bilaterally.

Table 1 summarizes her laboratory tests' results on admission. Briefly, leukocyte count was 4,810/*μ*L (neutrophil count 2,732/*μ*L); hemoglobin was 85 g/L; and platelet count was 30.0 × 10^4^/*μ*L. Among the serum biochemical parameters, C-reactive protein was 161.0 nmol/L; albumin was 21 g/L; blood urea nitrogen was 10.353 mmol/L; and creatinine was 499.5 *μ*mol/L. All other biochemical parameters were compatible with results generally observed in patients undergoing PD. Urinalysis showed 1+ proteinuria and 1+ hematuria. Her stool was positive for occult blood.

**Figure 1:**
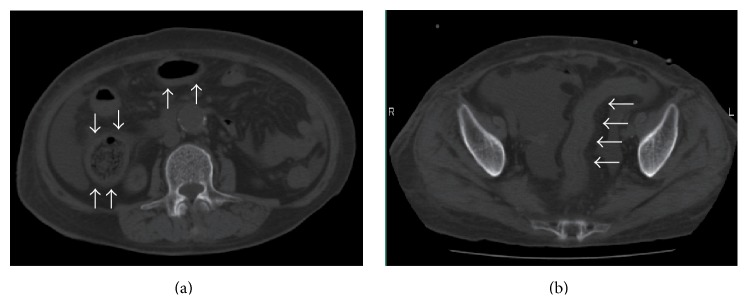
Computed tomography before treatment. Diffuse wall thickness of (a) the ascending colon and transverse colon and (b) the sigmoid colon (white arrows).

**Figure 2:**
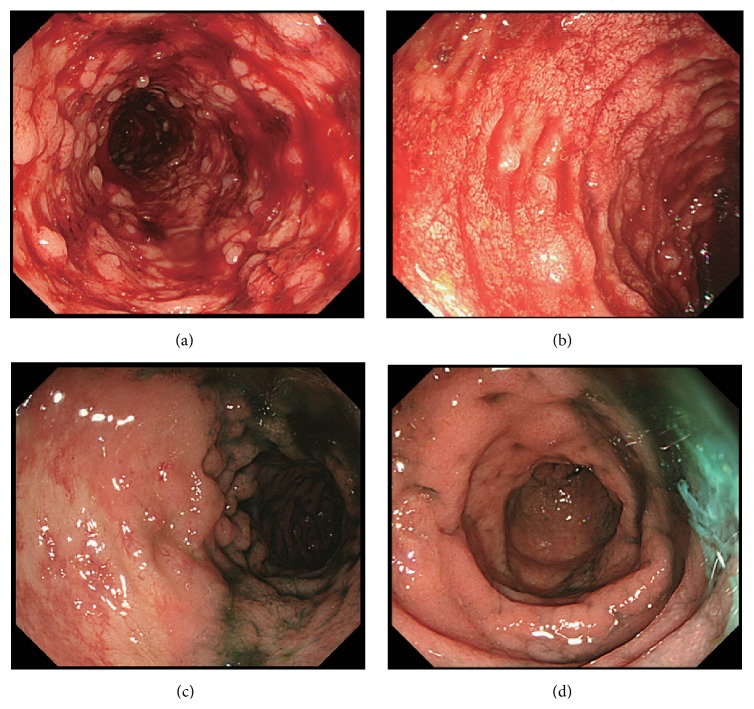
Total colonoscopy results before and after treatment. (a) Descending colon and (b) sigmoid colon before treatment, showing diffuse mucous erythema, spontaneous bleeding, and ulcerations. Disappearance of a vascular pattern was also observed. (c) Descending colon and (d) sigmoid colon after treatment, showing improvement of mucous inflammation.

Imaging modalities were used to determine the cause of bloody stool. Abdominal X-rays showed the disappearance of haustra coli throughout the entire descending colon. Computed tomography showed extensive wall thickness from the ascending colon to the rectum, without abscesses (Figure 1). Total colonoscopy was performed on the second day in hospital and it showed ulceration, the disappearance of a vascular pattern, spontaneous bleeding, and mucopus (Figures 2(a) and 2(b)). Biopsy specimens of the affected colon and rectum showed chronic active inflammation of the colonic and rectal mucosae, with crypt damage and goblet cell depletion. All of these findings were compatible with UC, resulting in a diagnosis of UC (pancolitis type, moderate-to-severe form, Mayo score: 3).

**Figure 3:**
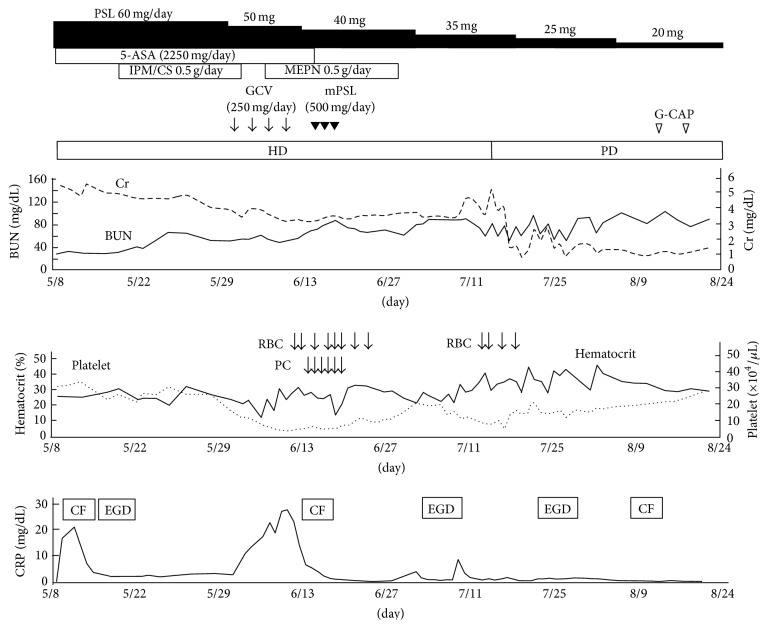
Clinical course. 5-ASA, 5-aminosalicylic acid; CF, colon fiber; BUN, blood urea nitrogen; Cr, creatinine; CRP, C-reactive protein; G-CAP, granulocyte and monocyte adsorption apheresis; GCV, ganciclovir; EGD, esophagogastroduodenoscopy; HD, hemodialysis; IPM/CS, imipenem/cilastatin; MEPN, meropenem; mPSL, methylprednisolone; PC, platelet concentrate; PD, peritoneal dialysis; PSL, prednisolone; RBC; red blood cell.

The clinical course of this patient is shown in Figure 3. She was treated based on the UC guideline used in Japan [6]. She was started on prednisolone (60 mg/day) and mesalazine (5-aminosalicylic acid, 2,250 mg/day) on the second hospital day. After starting prednisolone, serum inflammatory markers decreased and body temperature returned to normal, followed by decreased blood in the stool. The dose of prednisolone was gradually tapered, based on periodic clinical and colonoscopic findings. Total colonoscopy on day 93 showed improvement in mucous inflammation, as shown by the presence of a vascular pattern and a disappearance of spontaneous bleeding and ulceration (Mayo score: 1), indicating the effectiveness of steroid therapy (Figures 2(c) and 2(d)). She underwent five rounds of granulocyte and monocyte adsorption apheresis on day 95 to induce remission. Prednisolone was tapered to 17.5 mg/day on day 102 without aggravation of UC activity thereafter. Finally, she was transferred to a local hospital on day 109.

During her hospitalization, she experienced several complications associated with immunosuppressive treatment, including cytomegalovirus-related enteritis, drug-induced thrombocytopenia, fusobacterium-related sepsis, and bacteria-associated hemophagocytic syndrome. These conditions were successfully treated by blood transfusion, administration of ganciclovir and antibiotics, and methylprednisolone pulse therapy. Furthermore, renal replacement therapy was changed from PD to hemodialysis, because her activity of daily living deteriorated and steroid use increased the demand for higher clearance of uremic toxins.

## 3. Discussion

This report describes an elderly ESRD patient who presented with persistent fever and bloody diarrhea at the initiation of dialysis. Total colonoscopy revealed spontaneous bleeding, disappearance of a vascular pattern, and ulceration along the entire colon, with all of these findings compatible with UC. A series of immunosuppressive treatments attenuated the activity of UC and induced remission.

UC in dialysis patients has rarely been reported. By contrast, dialysis patients with Crohn's disease, another inflammatory bowel disease, have been occasionally reported [7, 8]. The reasons underlying the relative lack of reports on UC in dialysis patients are uncertain, although impaired immunological responses in patients with CKD may suppress UC activity [9, 10]. Alternatively, UC often affects relatively young patients, in contrast to our elderly patient [1, 2]. Because the UC activity that complicates CKD may be severe, these patients may finally require proctocolectomy, thus removing the causes of CKD and avoiding ESRD. In addition, dialysis patients with UC may go underreported. Thus, it is important to accumulate evidence on the appropriate treatments that can control UC disease activity in dialysis patients.

Fever is an important complication occurring in patients with ESRD at the initiation of dialysis. Several disease entities can induce fever at timing of dialysis initiation [11, 12], with tuberculosis being one of the most common causes, especially at the initiation of dialysis, in CKD patients [13]. In our patient, colonoscopy, which was performed to determine whether contraindication for PD was present, did not reveal any sign of UC at the time of dialysis initiation. Actually, the symptoms for the first two months after initiation of dialysis were nonspecific, including fever and fatigue, with no gastrointestinal symptoms and bloody diarrhea developing about three months after the onset of fever. Interestingly, a recent report described a patient with UC who presented with fever without gastroduodenal symptoms [14]. Although we could not completely rule out the possibility that fever at dialysis initiation was mediated by other causes, that finding and those shown here indicate the possibility of UC as a cause of fever even in dialysis patients without gastrointestinal symptoms.

Treatment of UC depends on disease activity [1, 2]. Because UC is an immunological disorder, immunosuppressive therapy is rational and effective [15]. However, the immune system in dialysis patients is already suppressed by the uremic milieu [9, 10]. Superimposition of immunosuppressive treatment on CKD patients can easily lead to opportunistic infections. For instance, glucocorticoids suppress a wide variety of immune components, causing devastating opportunistic infection. Actually, during treatment of UC, our patient developed cytomegalovirus infection and fusobacterium-related sepsis, requiring intensive treatment. Hence, treatment which has less impact on immune system may be more favorable especially in patients with ESRD patients who are prone to developing opportunistic infection.

As for the diagnosis and treatment in the present case report, several points should be described. First, total colonoscopy can aggravate UC especially when UC is very active. In those cases, sigmoidoscopy may be enough to diagnose and is considered. Second, treatment strategy and application of effective drugs are diverse depending on the countries and local guidelines [6]. Hence, our diagnostic and treatment strategy is one of the options among a variety of possibilities.

In summary, this report describes an elderly patient with ESRD who experienced an acute onset of UC presenting as bloody diarrhea. The patient was successfully treated by multimodal immunotherapy. The findings in our patient emphasize the importance of considering UC as a differential diagnosis in predialysis ESRD patients who present with fever and bloody diarrhea.

## Figures and Tables

**Table 1:** Results of laboratory tests conducted on admission.

Complete blood count		
Hemoglobin	85	g/L
Hematocrit	0.258	
White blood cells (×10^9^)	4.81	/L
Neutrophils (×10^9^)	2.732	/L
Platelets (×10^9^)	30.0	/L
Serum biochemistry		
Total protein	53	g/L
Albumin	21	g/L
Urea nitrogen	10.4	*μ*mol/L
Creatinine	499.5	*μ*mol/L
Uric acid	446.1	*μ*mol/L
Total bilirubin	6.84	*μ*mol/L
Glucose	7.992	mmol/L
C-reactive protein	161.0	nmol/L
Calcium	2.0	mmol/L
Phosphorus	1.0	mmol/L
Sodium	134	mmol/L
Potassium	3.7	mmol/L
Chloride	95	mmol/L
AST	12	U/L
ALT	7	U/L
Lactate dehydrogenase	173	U/L
Alkaline phosphatase	232	U/L
Amylase	109	U/L
Creatine kinase	14	U/L
Iron	5.19	*μ*mol/L
UIBC	4.30	*μ*mol/L
Ferritin	972.1	pmol/L
Total cholesterol	95	mmol/L
HDL-cholesterol	0.57	mmol/L
LDL-cholesterol	1.21	mmol/L
Triglyceride	1.50	mmol/L
Immunological studies		
Immunoglobulin G	15.94	g/L
Immunoglobulin A	3.24	g/L
Immunoglobulin M	0.79	g/L
C3	0.54	g/L
C4	0.23	g/L
CH50	60	U/L
Rheumatoid factor	8	U/mL
Anti-nuclear antibody	(—)	
Anti-HBs antigen	(—)	
Anti-HBs antibody	(—)	
Anti-HCV antibody	(—)	
Endocrinological studies		
Plasma cortisol	441.4	nmol/L
Plasma ACTH	12.8	mmol/L
Serum TSH	2.43	*μ*U/mL
Serum-free T4	18.4	pmol/L
Serum tumor markers		
CEA	1.3	*μ*g/L
CA19-9	7.3	U/mL

ACTH, adrenocorticotropic hormone; ALT, alanine aminotransferase; AST, aspartate aminotransferase; C, complement; CA19-9, carbohydrate antigen 19-9; CEA, carcinoembryonic antigen; HBs, hepatitis B surface; HCV, hepatitis C virus; HDL, high density lipoprotein; LDL, low density lipoprotein; UIBC, unsaturated iron binding capacity; TSH, thyroid stimulating hormone.

## References

[1] Ordás I., Eckmann L., Talamini M., Baumgart D. C., Sandborn W. J. (2012). Ulcerative colitis. *The Lancet*.

[2] Feuerstein J. D., Cheifetz A. S. (2014). Ulcerative colitis: epidemiology, diagnosis, and management. *Mayo Clinic Proceedings*.

[3] Lissner D., Siegmund B. (2013). Ulcerative colitis: current and future treatment strategies. *Digestive Diseases*.

[4] Ambruzs J. M., Walker P. D., Larsen C. P. (2014). The histopathologic spectrum of kidney biopsies in patients with inflammatory bowel disease. *Clinical Journal of the American Society of Nephrology*.

[5] Tokuyama H., Wakino S., Konishi K., Hashiguchi A., Hayashi K., Itoh H. (2010). Acute interstitial nephritis associated with ulcerative colitis. *Clinical and Experimental Nephrology*.

[6] Matsuoka K., Hibi T. (2013). Treatment guidelines in inflammatory bowel disease: the Japanese perspectives. *Digestive Diseases*.

[7] Chiba M., Tsuda S., Tsuji T. (2014). Crohn's disease successfully treated with infliximab in a patient receiving hemodialysis: case report and review of the literature. *Medicine*.

[8] Kume K., Yamasaki M., Yoshikawa I., Harada M. (2011). Infliximab treatment in a patient with Crohn's disease on haemodialysis. *Colorectal Disease*.

[9] Hauser A. B., Stinghen A. E. M., Kato S. (2008). Characteristics and causes of immune dysfunction related to uremia and dialysis. *Peritoneal Dialysis International*.

[10] Kato S., Chmielewski M., Honda H. (2008). Aspects of immune dysfunction in end-stage renal disease. *Clinical Journal of the American Society of Nephrology*.

[11] Hirschmann J. V. (1997). Fever of unknown origin in adults. *Clinical Infectious Diseases*.

[12] Arnow P. M., Flaherty J. P. (1997). Fever of unknown origin. *The Lancet*.

[13] Hussein M. M., Mooij J. M., Roujouleh H. (2003). Tuberculosis and chronic renal disease. *Seminars in Dialysis*.

[14] Shpilberg R., Hadjiyiannis D., Khan S. A. (2012). Ulcerative colitis presenting as pyrexia of unknown origin (PUO) without bowel symptoms. *Clinical Medicine*.

[15] Monteleone G., Pallone F., MacDonald T. T. (2011). Emerging immunological targets in inflammatory bowel disease. *Current Opinion in Pharmacology*.

